# Getting the invite list right: a discussion of sepsis severity scoring systems in severe complicated intra-abdominal sepsis and randomized trial inclusion criteria

**DOI:** 10.1186/s13017-018-0177-2

**Published:** 2018-04-06

**Authors:** Matti Tolonen, Federico Coccolini, Luca Ansaloni, Massimo Sartelli, Derek J. Roberts, Jessica L. McKee, Ari Leppaniemi, Christopher J. Doig, Fausto Catena, Timothy Fabian, Craig N. Jenne, Osvaldo Chiara, Paul Kubes, Yoram Kluger, Gustavo P. Fraga, Bruno M. Pereira, Jose J. Diaz, Michael Sugrue, Ernest E. Moore, Jianan Ren, Chad G. Ball, Raul Coimbra, Elijah Dixon, Walter Biffl, Anthony MacLean, Paul B. McBeth, Juan G. Posadas-Calleja, Salomone Di Saverio, Jimmy Xiao, Andrew W. Kirkpatrick, From the Closed Or Open after Laparotomy (COOL) for Source Control in Severe Complicated Intra-Abdominal Sepsis Investigators

**Affiliations:** 1 Department of Abdominal Surgery, Abdominal Center, University of Helsinki and Helsinki University Central Hospital, Helsinki, Finland; 2 Emergency and Trauma Surgery Department, Bufalini Hospital, Cesena, Italy; 3 Unit of General and Emergency Surgery, Bufalini Hospital of Cesena, Cesna, Italy; 4 Department of Surgery, Macerata Hospital, Macerata, Italy; 5 Department of Surgery, University of Calgary, Calgary, Alberta Canada; 6 Regional Trauma Services, Foothills Medical Centre, Calgary, Canada; 7 Departments of Critical Care Medicine and Community Health Sciences, Cumming School of Medicine, University of Calgary, Calgary, Canada; 8 Emergency Surgery Department, Parma University Hospital, Parma, Italy; 9 University of Tennessee Health Sciences Center, Memphis, TN USA; 10 Department of Critical Care Medicine, University of Calgary, Calgary, Alberta Canada; 11 General Surgery and Trauma Team Niguarda Hospital Milano, Milan, Italy; 12 Phoebe and Joan Snyder Institute for Chronic Diseases, University of Calgary, Calgary, Canada; 13 Departments of Physiology and Pharmacology Cumming School of Medicine, University of Calgary, Calgary, Canada; 14 Rambam Health Care Campus, Haifa, Israel; 15 Division of Trauma Surgery, University of Campinas, Campinas, SP Brazil; 16 Trauma/Acute Care Surgery and Surgical Critical Care, University of Campinas, Campinas, Brazil; 17 Department of Surgery, R Adams Cowley Shock Trauma Center, University of Maryland School on Medicine, Baltimore, MD USA; 18 Letterkenny University Hospital, Donegal Clinical Research Academy, Donegal, Ireland; 19 Trauma and Critical Care Research, University of Colorado, Denver, CO USA; 20 Department of Surgery, Jinling Hospital, Medical School of Nanjing University, Nanjing, China; 21 Acute Care, and Hepatobiliary Surgery, and Regional Trauma Services, University of Calgary, Calgary, Alberta Canada; 22 Riverside University Health System Medical Center, Moreno Valley, USA; 23 Loma Linda University School of Medicine, Loma Linda, CA USA; 24 Surgery, Oncology, and Community Health Sciences, City Wide Section of General Surgery, University of Calgary, Calgary, Alberta Canada; 25 Trauma and Acute Care Surgery, Scripps Memorial Hospital La Jolla, La Jolla, California USA; 26 Division of General Surgery Foothills Medical Centre, Department of Surgery, University of Calgary, Calgary, Canada; 27 The Trauma Program, University of Calgary, Calgary, Alberta Canada; 28 Cambridge University Hospitals NHS Foundation Trust, Addenbrooke’s Hospital, Cambridge, UK; 29 EG23 Foothills Medical Centre, Calgary, Alberta T2N 2T9 Canada

**Keywords:** Intra-abdominal sepsis, Septic shock, Risk stratification, Randomized controlled trial, Trial methodology, Organ dysfunction, Epidemiology

## Abstract

**Background:**

Severe complicated intra-abdominal sepsis (SCIAS) is a worldwide challenge with increasing incidence. Open abdomen management with enhanced clearance of fluid and biomediators from the peritoneum is a potential therapy requiring prospective evaluation. Given the complexity of powering multi-center trials, it is essential to recruit an inception cohort sick enough to benefit from the intervention; otherwise, no effect of a potentially beneficial therapy may be apparent. An evaluation of abilities of recognized predictive systems to recognize SCIAS patients was conducted using an existing intra-abdominal sepsis (IAS) database.

**Methods:**

All consecutive adult patients with a diffuse secondary peritonitis between 2012 and 2013 were collected from a quaternary care hospital in Finland, excluding appendicitis/cholecystitis. From this retrospectively collected database, a target population (93) of those with either ICU admission or mortality were selected. The performance metrics of the Third Consensus Definitions for Sepsis and Septic Shock based on both SOFA and quick SOFA, the World Society of Emergency Surgery Sepsis Severity Score (WSESSSS), the APACHE II score, Manheim Peritonitis Index (MPI), and the Calgary Predisposition, Infection, Response, and Organ dysfunction (CPIRO) score were all tested for their discriminant ability to identify this subgroup with SCIAS and to predict mortality.

**Results:**

Predictive systems with an area under-the-receiving-operating characteristic (AUC) curve > 0.8 included SOFA, Sepsis-3 definitions, APACHE II, WSESSSS, and CPIRO scores with the overall best for CPIRO. The highest identification rates were SOFA score ≥ 2 (78.4%), followed by the WSESSSS score ≥ 8 (73.1%), SOFA ≥ 3 (75.2%), and APACHE II ≥ 14 (68.8%) identification. Combining the Sepsis-3 septic-shock definition and WSESSS ≥ 8 increased detection to 80%. Including CPIRO score ≥ 3 increased this to 82.8% (Sensitivity-SN; 83% Specificity-SP; 74%. Comparatively, SOFA ≥ 4 and WSESSSS ≥ 8 with or without septic-shock had 83.9% detection (SN; 84%, SP; 75%, 25% mortality).

**Conclusions:**

No one scoring system behaves perfectly, and all are largely dominated by organ dysfunction. Utilizing combinations of SOFA, CPIRO, and WSESSSS scores in addition to the Sepsis-3 septic shock definition appears to offer the widest “inclusion-criteria” to recognize patients with a high chance of mortality and ICU admission.

**Trial registration:**

https://clinicaltrials.gov/ct2/show/NCT03163095; Registered on May 22, 2017.

## Background

Sepsis is a complex and increasing global health problem [1–5]. International consensus currently uses the working definition of sepsis as life-threatening organ dysfunction caused by a dysregulated host response to infection [2]. The number of cases per year is estimated as approaching 18–19 million worldwide [4–6]. In the most severe cases, mortality rates approach 30–40% when shock is present [2, 7, 8], although may be 80% in the developing world [9]. When the focus of infection is located within the abdominal cavity, a particularly severe form of sepsis may result in association with the anatomy and physiology of the abdominal cavity and the viscera within [10, 11]. Cases of intra-abdominal sepsis (IAS) may be defined as complicated when the inflammation or contamination spreads beyond a single organ [12, 13]. Complicated IAS may also be considered severe complicated IAS (SCIAS) when organ dysfunction is present with a mortality rate of 10–30% or with a mortality rate of 40–70% [14, 15] when septic shock is present [2, 7, 16, 17].

Despite advances in diagnosis, surgery, and antimicrobial therapy, mortality rates associated with CIAS and IAS remain exceedingly high [18]. Despite appropriate therapy, progress to septic shock and multiple organ dysfunction driven by inflammation is common. Delayed or inadequate source control remains an independent predictor of mortality [19, 20]. However, recognizing “failed source control” [21, 22], from a self-propagating biomediator storm is often difficult or impossible without abdominal re-exploration (relaparotomy). At present, pharmacologic approaches are not the answer. Attempting to derive pharmacologic therapies for combating post-infective inflammation has proven an expensive and frustrating process [23]. Over 100 attempts at blocking single biological response mediators have failed to address the early cytokine storm of sepsis [24, 25]. A controversial, potentially morbid, potentially life-saving technique is the adoption of an open abdomen (OA) following source control laparotomy. Uncontrolled use of the OA following sepsis is increasingly being reported as another potentially desirable option for the sickest SCIAS patents [12, 21, 22, 26–28]. However, accepting the OA is either potentially a life-saving intervention or a morbid unnecessary procedure with increased risks of complications such as enterocutaneous fistulae [29, 30]; an adequately powered prospective randomized controlled trial is urgently required.

Given the complexity of adequately powering multi-center trials, it is essential to recruit an inception cohort of patients sick enough to benefit from the intervention; otherwise, no effect of a potentially beneficial therapy may be apparent. Many scoring systems have been proposed for use in predicting clinical outcomes in the critically ill. Potential systems that have been suggested include the Acute Physiology and Chronic Health Evaluation (APACHE II) [31–34], multiple organ failure (MOF) scores [35], P-POSSUM [32, 36], Therapeutic Intervention Scoring System (TISS-28) [37, 38], and the National Early Warning Score (NEWS) definitions of sepsis [39, 40]; some are more intended for sepsis specifically such as the Sepsis Severity Score [33] and those specifically intended to consider intra-abdominal pathology such as the Mannheim Peritonitis Index [41], the World Society of Emergency Surgery Sepsis Severity Score (WSESSSS) [7], and even systems intended for pancreatitis such as the Ranson [42, 43] and Imrie [43] scores [44]. However, none is currently accepted as being ideal for predicting outcomes in SCIAS [7]. We thus conducted an evaluation of the abilities of recognized predictive systems for clinical outcomes in SCIAS to detect patients of interest using an existing IAS database.

## Methods

A retrospectively collected database of SCIAS cases was created at a quaternary care hospital in Helsinki, Finland. This database enrolled all consecutive adult patients with a diffuse secondary peritonitis between 2012 and 2013, although cases of appendicitis or cholecystitis were excluded. The institutional human research review committee approved the study design, and as it was an observational retrospective cohort study, neither informed consent nor ethics committee’s approval was required. An attribute of this dataset was that intraoperative evaluations for development of organ dysfunctions were performed. Although this data has been previously published [45], for the current project the original data was revisited and updated regarding to new Sepsis-3 definitions of organ dysfunction. The demographics included in this database have been previously described and were sufficient to allow calculation of the Mannheim Peritonitis Index (MPI), WSESSSS (Table 1), Calgary Predisposition, Infection, Response, and Organ Dysfunction (CPIRO) (Table 2), Acute Physiology and Chronic Health Evaluation (APACHE) II, and the consensus definitions and quick SOFA (qSOFA) score of the Sepsis-3 International Consensus Definitions. From this database, a cohort of patients who either died or were admitted to the intensive care unit were selected. Thereafter, the performance metrics of these putative predictive and scoring systems were tested (using a number of varying thresholds within each system where appropriate) for their discriminant ability to identify SCIAS and, thereafter, predict ICU admission or 30-day mortality. To assess whether utilizing combinations of scoring systems provided additive predictive power, the performance of combinations of systems was also calculated. This was done through simple mathematical addition of patient of interest recognition. Potential combinations to test were selected based on their being practically usable by on-call clinicians without retrospective data or extensive laboratory results, an acceptable AUROC (> 0.80) in our analysis reflecting a practical combination of scores in terms of highest sensitivity with a reasonable specificity. Analyses were performed using SPSS© Statistics version 22 for Mac (IBM©, Armonk, NY, USA). Sensitivity and specificity were calculated for each prognostic system. Receiving operating characteristics (ROC) curves were plotted and area under curve (AUC) calculated with 95% confidence interval (CI).

**Table 1:** World Society of Emergency Surgery Sepsis Severity Score for complicated intra-abdominal infections

Clinical conditions at admission	
Sepsis with organ dysfunction at admission	3 points
Septic shock (acute circulatory failure characterized by persistent arterial hypotension) requiring vasopressor agents	5 points
Setting of acquisition	
Healthcare-associated infection	2 points
Origin of intra-abdominal infection	
Colonic non-diverticular perforation peritonitis	2 points
Small bowel perforation peritonitis	3 points
Diverticular diffuse peritonitis	2 points
Post-operative diffuse peritonitis	2 points
Delay in source control	
Delayed initial intervention (pre-operative duration of peritonitis (localized or diffuse) > 24 h	3 points
Risk factor	
Age > 70	2 points
Immunosuppression (chronic glucocorticoids, immunosuppressive agents, chemotherapy, lymphatic disease, virus)	3 points

Reproduced from Sartelli et al [7]

**Table 2:** Calgary Predisposition, Infection, Response, and Organ Dysfunction (CPIRO)

Score	Variable	Point
Predisposition	Age > 65 years	1
	Comorbidities	1
Response	Leukopenia	1
	Hypothermia	1
Organ dysfunction	Cardiovascular dysfunction	1
	Respiratory dysfunction	1
	Renal dysfunction	1
	CNS dysfunction	1
Total		8

Table reproduced from Posadas-Calleja et al. [57]

## Results

In the original data set, there were 223 patients. Of these patients, 33 (13.5%) died within 30-days and 72 (32.2%) were admitted to the ICU. The target group of interest, with either 30-day mortality or ICU admission, constituted 93 patients with a 22% mortality. The majority (88%) of this group stayed in ICU more than 3 days and had a mean highest SOFA score of 7.9 [median 8; IQR 5–10]. Accounting for those that died in ICU, the mean length of ICU stay was 7.8 days [median 5.0; IQR 3–8.75], with 90% staying 3 or more days.

**Fig. 1:**
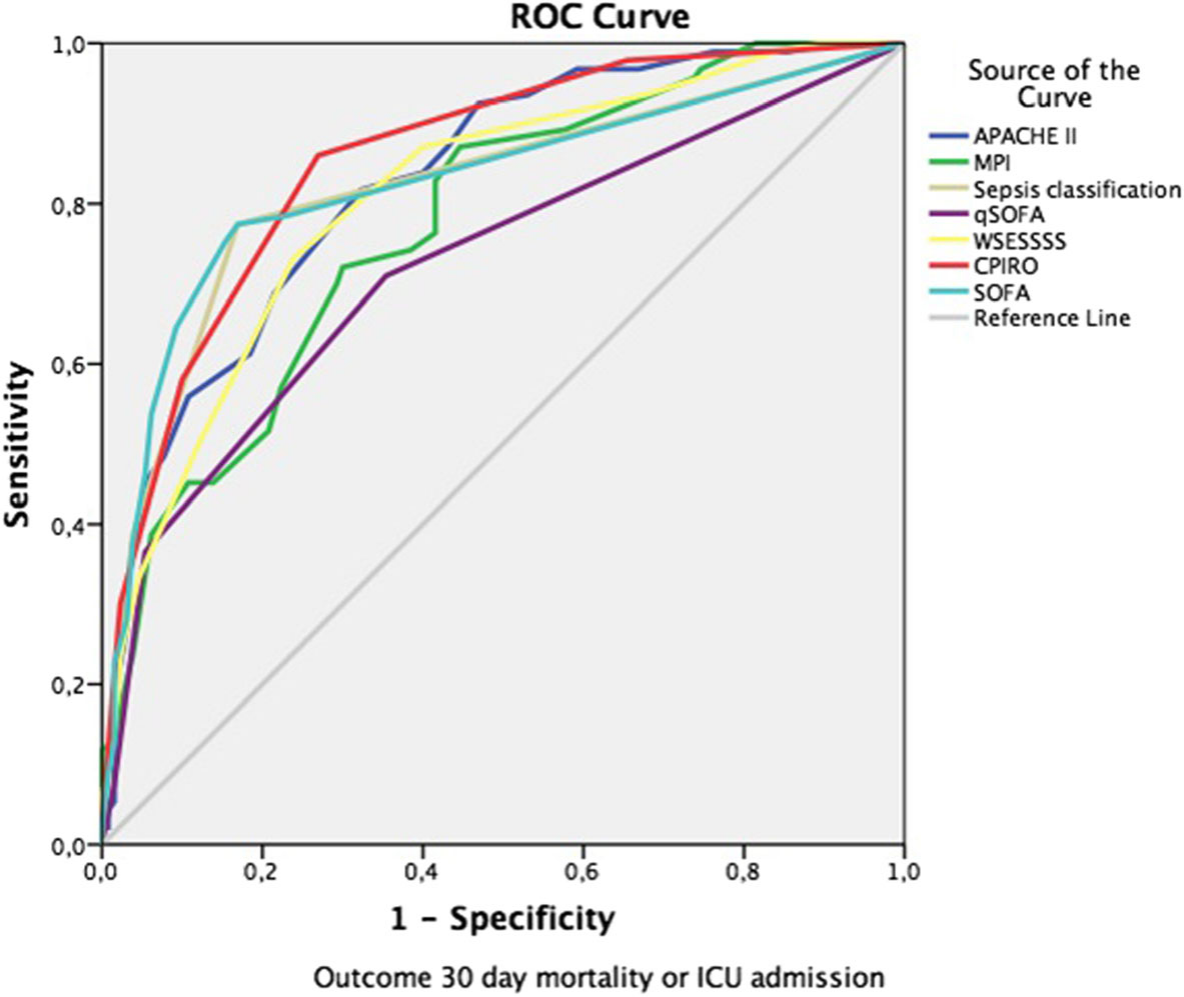
Area under the receiver-operating curve (AUC) for candidate scoring systems considering recruitment population of interest with ICU Admission or mortality

**Fig. 2:**
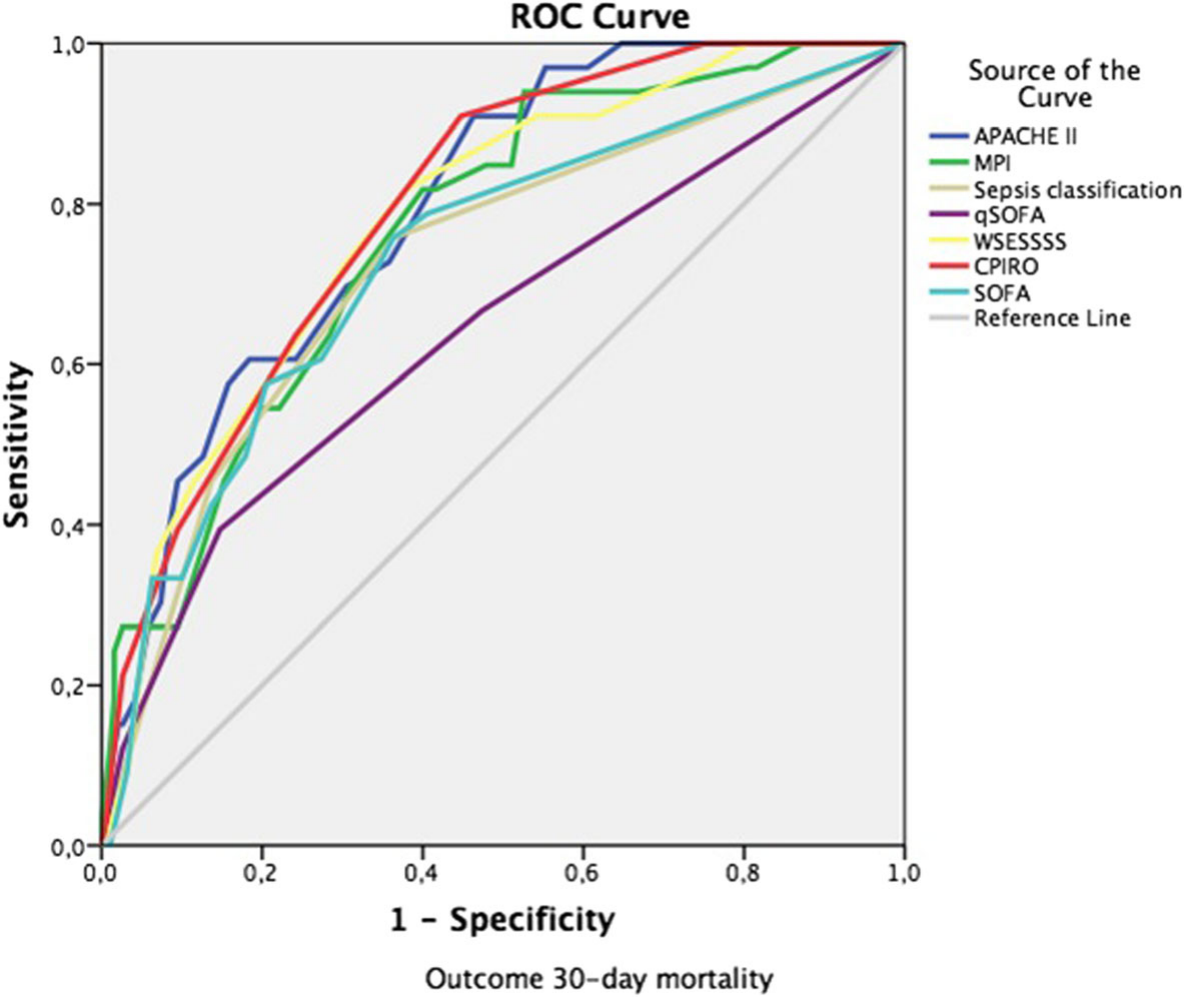
Area under the receiver-operating curve (AUC) for candidate scoring systems considering recruitment population of interest with mortality only

Overall predictive rates were tested for different threshold values of the scoring systems: qSOFA ≥ 2; SOFA 2, 3, 4 and Sepsis-3 septic shock definition; MPI ≥ 30, 32, 34; APACHE II ≥ 12, 14, 16; WSES ≥ 8, 9, 10; CPIRO ≥ 3, 4 (Table 3). In addition, the combined predictive capability of using the scores together was also tested and are reported in Table 4. Systems with a good (AUC > 0.8) performance included the SOFA, Sepsis-3 sepsis classification, APACHE II, WSESSSS, and CPIRO scores and overall; the greatest AUC was for the CPIRO score regardless if the consideration was of patients requiring ICU admission and dying (Fig. 1) or just with mortality (Fig. 2).

**Table 3:** Predictive capabilities of potential COOL study sepsis and critical illness scoring systems using Helsinki outcomes data

System	Identified	Outcome mortality	Sensitivity (%)	Specificity (%)	AUC^b^	95% CI
qSOFA ≥ 2	34 (36.6%)	13 (32%)	37	95	0.723	[0.653–0.792]
SOFA ≥ 2	72 (77.4%)	25 (27%)	77	83	0.825	[0.766–0.885]
SOFA ≥ 3	70 (75.3%)	24 (27%)	75	85		
SOFA ≥ 4	60 (64.5%)	20 (28%)	65	91		
Septic shock^a^	36 (38.7%)	15 (37%)	39	96	0.82	[0.761–0.88]
MPI ≥ 30	48 (51.6%)	21 (28%)	51	79	0.774	[0.713–0.835]
MPI ≥ 32	42 (45.2%)	18 (32%)	45	89		
MPI ≥ 34	22 (23.7%)	9 (33%)	24	96		
APACHE II ≥ 14	64 (68.8%)	24 (26%)	69	78	0.828	[0.775–0.881]
APACHE II ≥ 16	52 (55.9%)	20 (30%)	56	89		
APACHE II ≥ 18	42 (45.2%)	19 (39%)	45	95		
WSESSSS ≥ 8	68 (73.1%)	27 (27%)	73	76	0.809	[0.752–0.866]
WSESSSS ≥ 9	58 (62.4%)	24 (29%)	62	82		
WSESSSS ≥ 10	47 (50.5%)	20 (32%)	51	88		
CPIRO ≥ 3	54 (58.1%)	21 (31%)	58	90	0.856	[0.806–0.905]
CPIRO ≥ 4	28 (30.1%)	13 (42%)	30	98		

Ninety-three patients were selected out of the database based on 30-day mortality or ICU admission

^a^AUC is for sepsis classification according to Sepsis-3 consensus definitions

^b^Only one area under the curve (AUC) was calculated for each scoring system without thresholds within

**Table 4:** Combined predictive capabilities of potential COOL study sepsis and critical illness scoring systems using Helsinki outcomes data

System	Identified	Outcome mortality	Sensitivity (%)	Specificity (%)
Septic shock OR CPIRO ≥ 3	57 (61.3%)	21 (30%)	61	90
Septic shock OR CPIRO ≥ 4	42 (45.2%)	17 (35%)	45	95
SOFA ≥ 4 OR CPIRO ≥ 3	65 (69.9%)	22 (27%)	70	87
SOFA >4 (4 OR greater here) ≥ OR WSESSSS ≥ 8	78 (83.9%)	28 (25%)	84	75
Septic shock OR WSESSSS ≥ 8	74 (79.6%)	28 (26%)	80	75
Septic shock OR CPIRO ≥ 4 OR WSES ≥ 8	74 (79.6%)	28 (26%)	80	75
Septic shock OR CPIRO ≥ 3 OR WSES ≥ 8	77 (82.8%)	29 (26%)	83	74
Septic shock OR SOFA ≥ 4	60 (76.3%)	20 (28%)	65	91
Septic shock OR SOFA ≥ 4 OR WSES ≥ 8	78 (83.92%)	28 (25%)	84	75

Ninety-three patients were selected out of the database based on 30-day mortality or ICU admission

While selecting all patients with diffuse secondary peritonitis would yield greater enrollment, the disease severity would include patients with lower mortality that may not benefit from OA therapy. Thus, the most efficient identification rates of the desired cohort for a single system was a SOFA score ≥ 2 with 78.4% identification, followed by SOFA ≥ 3 with 75.2%, WSESSSS score ≥ 8 with 73.1%, and the APACHE II ≥ 14 with 68.8% identification (Table 1). Combining predictive systems together improved the identification rates (Table 4). Combining the Sepsis-3 septic shock definition with the WSESSS criteria increased detection to the highest rate of 79.6%, a rate that was not changed with the further addition of patients detected by a CPIRO score of ≥ 4 (Table 1). However, when the CPIRO was utilized with a threshold of 3 or greater, for an inclusive criteria of Sepsis-3 septic shock OR WSESSS ≥ 8 or CPIRO ≥ 3, this resulted in a detection rate of the desired population of 82.8%, with a sensitivity of 83% and a 74% specificity for detection of a population with the mortality rate remaining 26%. The other highest combination of scoring systems was combining a SOFA ≥ 4 with WSESSSS ≥ 8 which had a slightly higher detection rate of 83.9%, with 84% sensitivity and 75% specificity in a population with 25% mortality. This detection was unchanged in either direction by adding the criterion of septic shock.

## Discussion

The Closed or Open after Laparotomy (COOL) for Source Control in Severe Complicated Intra-abdominal Sepsis Trial (https://clinicaltrials.gov/ct2/show/NCT03163095) is a prospective multi-institutional worldwide study examining outcomes in those managed with primary fascial closure or OA in SCIAS [46]. To properly power this trial, it is necessary to utilize optimal and validated scoring tool(s) to identify surgical patients with SCIAS at high risk of death requiring ICU care early in their hospital course, typically prior to ICU admission. Such potential tool(s) should be easy to use and functional while still in the operating room prior to potential formal primary fascial closure. A remarkable variety of potential scoring systems for predicting outcomes in relation to septic populations have developed over time leading to a somewhat discrepant epidemiological picture that is compounded by the variety of populations and health care settings in which they have been studied. Systems have been used for a variety of reasons including quality assessment and audit, epidemiological reporting and comparison, study recruitment and analysis, and outcome clinical predictions [7, 28, 31–37, 39–44].

When interrogated against a cohort of SCIAS patients recruited from an advanced health-care system, each predictive model performed with different attributes. Even considering a specific scoring tool, the specific tool may have varying sensitivity and specificity for identifying patients of interest depending on the threshold value of the tool selected. Thus, no one scoring system behaved perfectly, and all appear to be largely dominated by organ dysfunction with a modest increase in detection provided by the inclusion of further patient characteristics some of which may not be readily available pre-operatively before potential surgery and admission to ICU. Nonetheless in this population, patients identified by the Sepsis-3 septic shock definition in addition to the WSESSS criteria with a score of 8 or greater had a detection rate of 80%. Adding the potential increased detection of the CPIRO score ≥ 3 increased the detection rate to 83%. Thus, the COOL investigators decision to include any of the three identifying criteria of Sepsis-3 septic shock criterion, WSESSSS ≥ 8, or CPIRO ≥ 3 seems statistically justified based on this analysis.

The strengths of the Helsinki dataset are that it involves an inception cohort exclusively of patients with IAS. A methodological concern involves the degree to which scores developed for septic patients with a wide range of precipitating causes will specifically identify patients with SCIAS. It is relevant to compare the specific experiences of the Helsinki data with evaluations in other general septic cohorts and especially cohorts of those with SCIAS. Although sepsis has been thoroughly studied in general critical care unit populations, accurate data collection has been less well studied outside of the ICU and early in intra-abdominal sepsis populations [39]. Thus, the literature does not support any one scoring or predictive systems as being established for use in SCIAS (Table 5).

**Table 5:** Previous assessments of predictive capabilities of sepsis scoring systems

Population	Inclusion criteria	Scores tested	Outcome 1	Outcome 2 (if applicable)
Ward or ED (*n* = 380) Szakmany 2017 [39]	NEWS ≥ 3		30-day mortality	Organ dysfunction
		Sepsis-1	68% SENS	
		Sepsis-3	86% SENS	
		qSOFA ≥ 2	22% SENS	26% SENS
		SOFA	0.70 AUC	86% SENS
		SIRS		26% SENS
		Sepsis-1 (severe)	92% SENS	
		NEWS > 6	41% SENS	36% SENS
			0.59 AUC	
Post-operative ICU patients [44] (*n* = 50)	Peritonitis + IAS requiring ICU		Multivariate Prediction of in-hospital death	
		APACHE II	HR 6.7 [95CI 2.7–17]	
		MPI	HR 9.8 095CI 1.3–73]	
		SAPS	NS	
		SSS	NS	
		MOF	NS	
		Ranson	NS	
		Imrie	NS	
			Predictions of death (day 1 SICU)	
Post-operative SICU patients (*n* = 145) Delibegovic 2011 [37]	Non-traumatic secondary peritonitis requiring laparotomy	TISS-28	AUC 0.87	
		APACHE II	AUC 0.86	
		MODS	AUC 0.83	
		SAPS	AUC 0.83	
		MPI	AUC 0.72	
		SSS	AUC 0.70	
Abdominal septic shock in ICU (*n* = 382) Hanisch 2011 [47]	Sepsis-1 criteria [61] for abdominal septic shock	SOFA	Death prediction first 3 ICU days	0.54 AUC
		APACHE II		0.52 AUC
		SAPS		0.52 AUC
		MODS		0.52 AUC
IAS pts requiring SCL (*n* = 211) Chan [48]	SCL and severe sepsis or septic shock as per Sepsis-1 (61)	APACHE-IV	Predicted mortality rate	0.67 AUC
IAS pts requiting re-laparotomy (*n* = 34) Das [32]	Secondary peritonitis	APACHE-II	Hospital mortality prediction	0.958 AUC
		SAPS-II		0.955 AUC
		P-POSSUM		0.931 AUC
IAS pts undergoing SCL (*n* = 221) Van Ruler [50]	APACHE > 10		Mortality	Need for Re-Laparotomy
		APACHE-II	0.74 AUC	0.49 AUC
		SAPS-II	0.80 AUC	0.56 AUC
		MPI	0.60 AUC	0.52 AUC
		SOFA	0.72 AUC	0.55 AUC
		MODS	0.76 AUC	0.55 AUC
		APS	0.68 AUC	.60 AUC

*ED* emergency department; *NEWS* National Early Warning Score; *Sepsis 1* use of the First Sepsis Consensus Definitions [61]; *SENS* sensitivity; *Sepsis-3* use of the Third Sepsis Consensus Definitions [2]; *qSOFA* quick SOFA score as per Sepsis-3 [2]; *SOFA* Sepsis-related Organ Failure Assessment [62]; *AUC* area under the curve of a receiver/operator curve; *SIRS* Systemic Inflammatory Response Syndrome Criteria as per Sepsis-1 consensus definitions [61]; *APACHE II* Acute Physiology and Chronic Health Evaluation II [31]; *MPI* Mannheim Peritonitis Index [41]; *SAPS* Simplified Acute Physiology Score [33]; *SSS* Sepsis Severity Score [33]; *MOF* multiple organ failure [35]; *TISS-28* Therapeutic Intervention Scoring System [38]; *IAS* intra-abdominal sepsis; *APACHE-IV* Acute Physiology and Chronic Health Evaluation IV [63]; *SCL* Source Control Laparotomy; *SAPS-II* Simplified Acute Physiology Score-II [64]; *P-POSSUM* Physiological and Operative Severity Score for the enumeration of Mortality and morbidity [36]; *APS* physiological part extracted from APACHE II [31]; *MODS* Multiple Organ Dysfunction Syndrome

For practical discussion, it will be important to detect potential enrollees on the ward and in the emergency department prior to post-surgical ICU admission. A previous determination of predictive capabilities of patients on the ward or emergency department with suspected infection examined the Sepsis-1, Sepsis-3, and NEWS definitions of sepsis [39, 40]. Szakmany and colleagues concluded that the Sepsis-3 definition identified patients with the highest risk. SOFA score and NEWS were better predictors of poor outcome, while the SOFA score appeared to be the best tool for identifying patients with high risk of death and sepsis-induced organ dysfunction [39]. Representative performance characteristics in this cohort with sepsis from all causes found that there was considerable overlap. Alternatively, application of SIRS-based criteria (Sepsis-1) did not identify 105 (27.3%) patients, all of whom had evidence of acute organ dysfunction [39]. This analysis again raised concerns about the performance of qSOFA as it only identified 13% of patients otherwise diagnosed with sepsis, missed 30% of those with organ dysfunction, and failed to predict mortality.

The COOL investigators were particularly interested in evaluations of scoring systems involving SCIAS populations. While there have been numerous scores that attempt to prognosticate outcomes for general septic populations, focused studies in CIAS are fewer. In 1997, Bosscha and colleagues, studying 50 patients, commented that there was no ideal and accepted predictive scoring system for IAS and only the MPI and APACHE II scores contributed independently to mortality prediction [44]. A comparison of the attributes of APACHE II, SAPS, sepsis score, MOF score, and TISS-28 in 145 patients with secondary peritonitis also concluded that the APACHE II and TISS-28 were significantly better than other systems and specifically criticized the power of the MPI [37]. They also specifically recommended that combining scoring systems together should be the standard classification system for grading severity of IAS [44]. Hanisch and colleagues analyzed 382 patients with “abdominal septic shock” in 2011 using the SOFA, APACHE II, SAPS, and MODS score and concluded that it was impossible to predict individual patient outcomes with any certainty and that the APACHE II performed the worst [47]. Concerns about APACHE, even the newest proprietary APACHE IV, were recently repeated by Chan and colleagues in a 2016 retrospective cohort analysis of IAS patients. They commented that the APACHE scores might not accurately predict mortality in those requiring source control laparotomies as the post-operative trajectory might be greatly modulated by the surgical procedure [48]. Concurrently, in 2014 Das and colleagues evaluated the SAPS, APACHE-II, and P-POSSUM systems to identify high risk surgical patients with intra-abdominal sepsis and planned relaparotomy [32]. Contrary to these other experiences, the APACHE II score was the best at predicting mortality in this small series of 34 patients with a 21% mortality rate [32]. However, although considered a good marker, the APACHE II utility in peritonitis has been questioned because of the conundrum of using the APACHE II to evaluate interventions despite the fact that interventions might significantly alter many of the physiological variables required for its calculation [7]. The authors of the RELAP trial [49] evaluated the APACHE II, SAPS, MPI, MODS, SOFA, and acute part of the APACHE-II score and noted that none were of clinical value to predict patients with a need for relaparotomy for IAS control and modest abilities in predicting in-hospital mortality [50].

The Predisposition, Infection, Response and Organ dysfunction (PIRO) staging system was designed as a stratification tool to deal with the inherent heterogeneity of septic patients [51]. The concept dates from recommendations made in the 2001 International Sepsis Definitions Conference to improve the traditional classification of sepsis [52, 53]. PIRO systems incorporate assessment of premorbid baseline susceptibility (predisposition), specific disorders responsible for illness (infection), responses of the host, and resulting degree of organ dysfunction. PIRO scores have been developed in patients with severe sepsis [54], community-acquired pneumonia (CAP) [55], and ventilator-associated pneumonia [56]. Evaluation in septic patients (25% intra-abdominal sepsis) in the emergency department suggested the PIRO score had a significantly improved AUC than both APACHE II and Mortality in the Emergency Department scores [51]. In Calgary, the CPIRO score showed consistent mortality discrimination outperforming both APACHE II and SOFA [57]. The mortality rate by CPIRO score was 37.6% for a CPIRO of 4 and 54.7% for a CPIRO of 5 during its development, and when tested with the Helsinki data, it had the highest AUC.

Another tool to potentially identify patients with intra-abdominal sepsis at a high risk of death is a World Society of Emergency Surgery Sepsis Severity Score of 8 points or more [7]. The World Society of Emergency Surgery (WSES) derived the WSESSSS from data and experience obtained from a global prospective observational study (CIAOW Study) that recruited patients in 132 medical institutions located in 54 countries [16, 17]. Seven hundred ninety-one patients (17.4%) were admitted in critical condition (septic shock or severe sepsis according to Sepsis-2 definitions [52]). The most significant variables, adjusted to clinical criteria, were used to create a severity score for patients with complicated intra-abdominal infections (cIAIs) including clinical conditions at admission (severe sepsis/septic shock), the origin of the cIAIs, the delay in source control, the setting of acquisition, and any risk factors such as age and immunosuppression. This predictive system carries the advantage of having been validated in a different worldwide population, giving great generalizability to the scoring system. In general, a score above 5.5 was the best predictor of mortality, but scores of 8 or more had a 41.7% mortality [7], very comparable to other groups of patients presenting with septic shock.

**Fig. 3:**
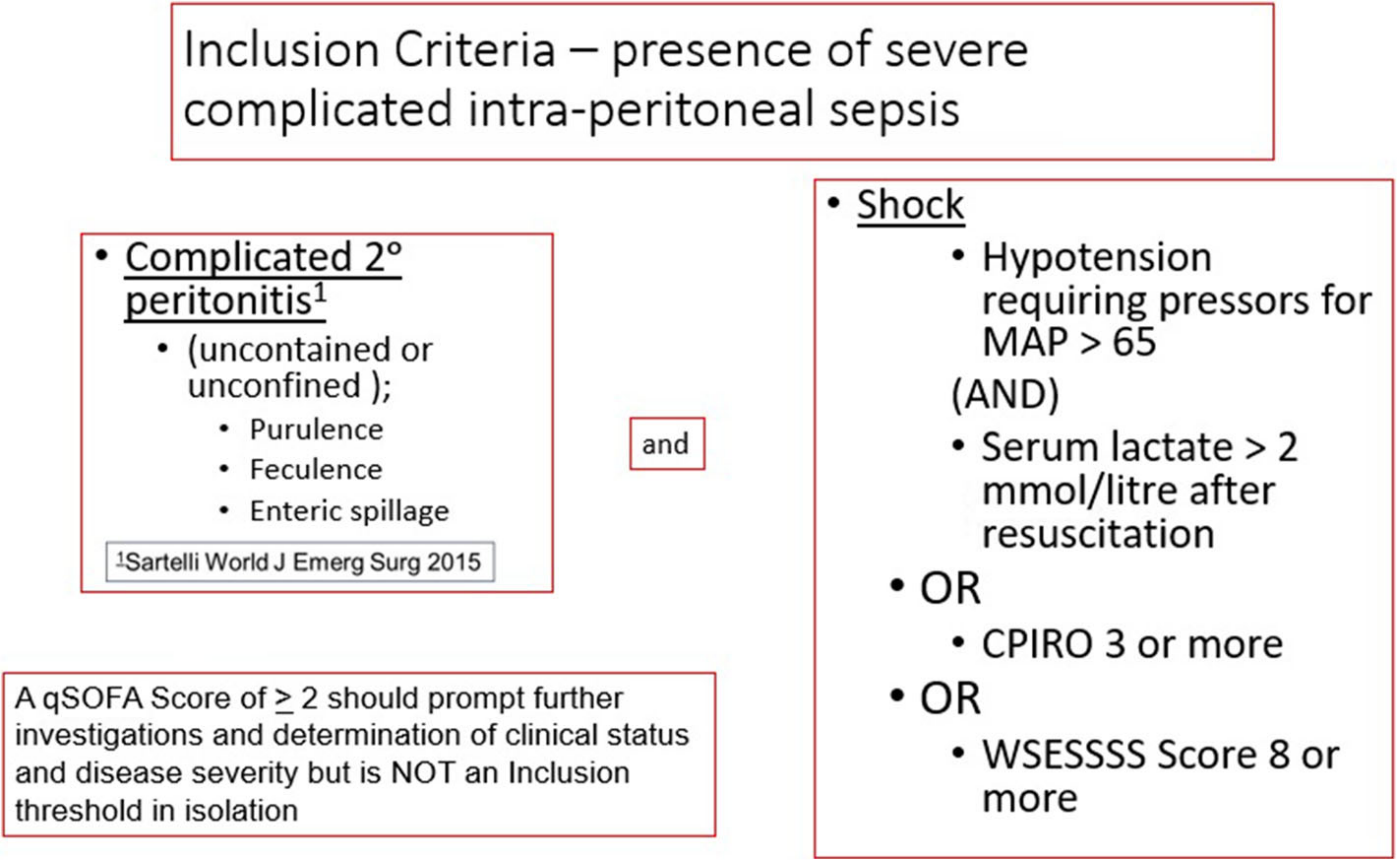
Parma inclusion criteria for the COOL study as adopted at the Advisory Panel Meeting Parma, Italy

Combining the formal SOFA score with the WSESSSS had a marginally higher detection rate than combining the WSESSS, CPIRO, and septic shock (83.9 versus 82.8%). Practical considerations that both the CPIRO and WSESSS were designed to be used early in sepsis and include expanded patient-specific criteria that do not require periods of observation undergoing critical care were felt to mitigate the marginally improved detection of alternatively using the SOFA as an early marker. The qSOFA was developed as a simple clinical criterion to identify patients with suspected infection who were likely to have poor outcomes, but it was also suggested that this might constitute entry criteria for clinical trials, which the COOL investigators strongly considered. However, the qSOFA actually had the lowest AUC of the systems formally tested and one of the lowest prediction rates in this population. This finding in conjunction with a growing opinion that the qSOFA may not be sensitive enough tool for its intended purpose [39, 58–60] led the COOL advisory panel to remove this inclusion criteria from the study protocol. Nonetheless, a triggering of qSOFA criteria in any patient with complicated IAS who may require operative source control should alert the caregivers to assess further whether critical features of SCIAS are present (Fig. 3).

## Conclusion

No one scoring system behaves perfectly, and all appear to be largely dominated by organ dysfunction. Utilizing both the CPIRO and WSESSSS scores in addition to the Sepsis-3 septic shock definition, combining the SOFA score with WSESSSS to detect seriously ill patients with IAS, offered the widest “net” to recognize patients with a high chance of mortality and ICU admission. Given practical considerations, utilizing septic shock, CPIRO, and WSESSS will form the basis of patient recruitment into the COOL study in an additive fashion wherein patients meeting any of the severity score criterion will be eligible (Fig. 2). The qSOFA score was considered insufficiently sensitive to serve as an eligibility criterion but will nonetheless remain useful in a preoperative setting to identify patients who require further evaluation, investigation, and care and might be eligible with further information. Overall, efforts to refine predictive scoring will benefit investigators looking to optimize inception cohorts among other scientists attempting to understand and treat SCIAS.
